# Near-death experiences and the importance of transparency in subjectivity, ontology and epistemology

**DOI:** 10.1093/braincomms/fcab304

**Published:** 2021-12-28

**Authors:** Tobias Kvist Stripp

**Affiliations:** University of Southern Denmark, J. B. Winsløvsvej 9, Odense 5000, Denmark

I read with great interest and diligence the paper from Peinkhofer *et al*.^1^ regarding how near-death experiences (NDEs) could be understood as the evolutionary product of death feigning (thanatosis). The authors provided this rather novel and interesting hypothesis regarding the evolutionary origin of NDEs, a topic that has most definitely received little attention in research. Could there be an adaptative or survival benefit biologically justifying the rich, subjective perceptions of the NDE? The authors of the paper provide a straightforward approach with a predefined methodology to test their hypothesis. But after this point, the argumentation and evidence brought forward by their quite rigorous approach, in my humble opinion, is less than compelling.

## Ontological and epistemological fallacies

The evidence that the authors provide does not justify the overall conclusion that NDEs primarily have a biological purpose and are indeed the evolutionary ‘upgrade’ of thanatosis. Ironically, the type of evidence brought forward as the basis of this conclusion is more or less identical with what is most criticized about the subjective or paranormal observations from NDE research, namely the evidence is anecdotal. This is also the case, e.g. with NDEs with veridical perceptions, which have not yet been demonstrated in a controlled prospective setup. These accounts, which surely challenge the reductionistic paradigm by indicating a consciousness outside of the brain, are often dismissed due to being anecdotes. I am not suggesting that NDEs and the anecdotes of veridical perceptions should be considered as a proof of non-local consciousness. I am simply pointing out an inconsistency in when and where the anecdotal evidence is accepted as the basis of scientific conclusions. This perhaps points to a reductionistic bias that should be reflected critically upon. An argument that might serve to highlight this bias appears in the first sentences of the abstract: ‘Near-death experiences are known from all parts of the world, various times and numerous cultural backgrounds. This universality suggests that near-death experiences may have a biological origin and purpose.’^1^ Such a statement is based on ontological materialistic assumptions. These are assumptions that the authors fail to mention or discuss, leading to a circular reasoning fallacy: since everything is only biology, NDEs have a biological purpose. I am not arguing against a commonality among all humans but suggesting that commonality may not necessarily amass solely to the biological components constituting the human body. We simply do not know all that ties us together. Furthermore, pure objectivity is, in many views, impossible, as there will always be some subjectivity and human decision in all research.^2^ Also, this subjectivity introduces a bias that one should reflect critically upon. Even the most common axioms of science are human constructs and should be treated as such.

## Discussion of the evidence provided

Work package 1 provides no surprises, as the authors diligently provide evidence that thanatosis and tonic immobility are well-preserved traits in humans. Furthermore, through rigorous search in both medical and non-medical research databases, the authors find, not surprisingly, that humans do indeed exhibit tonic immobility under the attack of a predator, for among other reasons, survival purposes (e.g. the Holocaust). They also provide evidence that NDEs do occur in life-threatening situations, which is in accord with the definition of an NDE and NDE research.^3^ The authors arbitrarily differentiate between NDEs occurring during assaults and by attack from ‘modern predators’, such as car accidents, and NDEs occurring during all other life-threatening situations, such as meningitis or cardiac arrest. They fail to define, discuss or reflect on the term ‘predator’. However, a quick lookup could provide a (biological) definition such as from the *Encyclopedia of Ecology* (2008): ‘Predation is the ecological process by which energy is transferred from living animal to living animal based on the behavior of a predator that captures and kills a prey before eating it.’^4^ In such a definition, cars would most certainly not fall under such a category of ‘modern predators’. The authors then pursue NDE reports that had occurred during tonic immobility in the face of a (modern) predator attack to link thanatosis to the evolution of NDEs. However, the authors failed to find any NDE accounts that were in fact reported in combination with tonic immobility or thanatosis.

On page 6, the authors discuss how tonic immobility or thanatosis may lead to a survival advantage only if some consciousness of the situation of attack is preserved, so that the unlikely opportunity of escape may be exploited instantaneously.^1^ It should be noted that near-death experiencers (NDErs) usually do not decide when to leave their NDE. In fact, many NDErs report that they did not want to leave their experience but were involuntarily forced to return to their body.^5^ More such inconsistencies are reported that are not in line with the actual phenomenology of NDEs. Similarly, it is long since it was shown that NDEs are not dreams or hallucinations, since NDEs are stringent in narrative, clear, can be remembered for decades and are most often deemed real or more real than everyday reality, while dreams or hallucinations are bizarre, difficult to remember clearly and are often deemed ‘unreal’ by the experiencers themselves.^3^

The authors also mention that the reason why some NDErs cannot fully express their NDE with common language must be that they are ‘less eloquent’. The authors then fail to refer to a study on how the eloquence of NDErs is associated with linguistic expression of their experience. If that is the case, that the authors have no such claim, this is a rather strong judgement to deal those having had life-changing experiences that they cannot precisely describe. In fact, some of the authors of the paper have contributed to develop the NDE-C scale,^6^ in which they have added a new item (NDE-C20): ‘You sense that the experience cannot be described adequately in words’—suggesting that ineffability is actually a common NDE phenomenon—and not one necessarily associated with low eloquence. Could it be that some of the phenomena of the NDE are outside understandings conveyed by a common language? Such phenomena require the analogies of myths and metaphors to hint at the true experience. Likewise, an experience such as holding your newborn child in your arms for the first time might be described as ‘breathtaking’—although it is rare that the parent stops breathing due to this.

While a rigorous methodology is warranted in science, rigorous reflection on practices, ontological presumptions, consistency and one’s own subjectivity is needed as well. As such, the acknowledging of a ‘subjective’ stance in relation to the research process and subject, and the reflection, reporting and transparency of this stance, could very well be the most ‘objective’ approach.^7^

## Conclusion

The paper by Peinkhofer *et al*. proposes an interesting perspective to NDE research and provides a novel hypothesis. Surely research into the possible (biological) benefits of NDEs is a warranted field of study, and the (biological) origin of the phenomena has yet to be uncovered. While endeavoring on such a journey, one should remember to reflect critically on one’s own subjectivity and stance. Also, as I have tried to outline without such reflection, logical and ontological fallacies risk biasing research. I hope that these comments may spark fruitful academic discussion to strengthen scientific practice—not only in regard to NDE research, but also with benefit to other scientific practices.

## Data availability

Data sharing is not applicable to this article as no new data were created or analysed.
